# Evaluation of Readmission Ink as a Marker for Dispersal Studies with the Oriental Fruit Fly, *Bactrocera dorsalis*

**DOI:** 10.1673/031.011.12501

**Published:** 2011-09-21

**Authors:** Karen M. Froerer, Steven L. Peck, Grant T. McQuate

**Affiliations:** ^1^ Department of Biology, Brigham Young University, Provo, UT 84602-5255; ^2^ USDA-ARS, US Pacific Basin Agricultural Research Center, 64 Nowelo Street, Hilo, HI 96720

**Keywords:** insect movement studies, invasive species, longevity, marking, mark-release-recapture

## Abstract

In this text we present a new marking dye, readmission ink, Blak-Ray, for the purpose of insect movement studies. The dye was tested in a controlled experiment with *Bactrocera dorsalis* (Hendel) (Diptera: Tephritidae) in anticipation of a long distance movement study planned for the following year with the same species. 700 individuals of both sexes were marked with the dye and placed in holding containers. Both the percentage of mortality and the ease of dye detection were monitored throughout a five-week period. Results showed minimal fly mortality and exceptional ease of dye detection.

## Introduction

Insect marking as a tool for studying population dynamics began in the early 20^th^ century; several materials and techniques have been tested since its initiation,including tags, paint, dye, genetic markings (Hagler and Jackson 2001), coding, dusts, radioisotopes, and elemental enrichment (Akey 1991). Each material or technique has differing degrees of effectiveness, and some have shown to be more suitable for specific insects over others (Hunt et al. 2000; Vilarinho et al. 2006). An effective marker is one that is durable, inexpensive, nontoxic, easy to apply and identify, and does not affect normal behavior, growth, fecundity, or life span (Akey 1991; Southwood and Henderson 2000; Hagler and Jackson 2001).

Depending on the marking technique used, identification of recaptured insects in a mark-release-recapture technique can be time consuming, especially when dealing with large numbers of wild specimens in the sample. In the use of DayGlo dye (DayGlo, www.dayglo.com), which is frequently used in fruit fly studies, the head of each captured fly must be excised and crushed with acetone in order to visually determine the presence or lack of the dye under blacklight (Peck and McQuate 2004; Southwood and Henderson 2000). In some cases, minute particles of DayGlo dye can be detected on the body, or in rare cases the ptilinum can be examined under a stereo microscope (McInnis et al. 2007). Another technique that has been used in tephritid fruit fly movement studies has involved marking adult flies with vertebrate protein with subsequent use of a sandwich enzyme-linked immunoabsorbent assay for marker detection (Hagler 1997; Peck and McQuate 2004). Although both of these techniques have proven effective, both are time consuming in cases where large numbers of samples need to be processed. A mark-release-recapture experiment was planned in order to assess the distance from which oriental fruit flies, *Bactrocera dorsalis* (Hendel) (Diptera: Tephritidae), might move relative to a suppression area. Because over two-hundred thousand marked flies were anticipated to be released in an environment with high oriental fruit fly population levels, the integration of a new marker requiring less processing time seemed appropriate. Blak-Ray (UVP, www.uvp.com) was chosen for testing. This dye is commonly used for re-admittance hand stamps in theme parks, dance clubs, and other venues. It is also popular as a quick drying agent, inhibiting false stamps being “shared” by paying clientele to non-paying clientele. In this paper, the effect of the dye on fly mortality and the persistence of the marker up to five weeks after marking was tested.

## Materials and Methods

In order to test the suitability of this dye for insect marking, oriental fruit flies were reared and irradiated to induce sterility by the USDA-ARS Pacific Basin Agricultural Research Center in Honolulu, HI, and sent to Hilo, HI. Thirty ml of pupae were then placed in separate 4.0 liter buckets with screen lids and held for adult emergence. When flies were two days old they were immobilized at 4° C, and a thin coat of ink was applied individually to the dorsum of 700 flies of each sex with a small paintbrush. While flies were individually marked to meet the goals of this experiment, spraying the flies using a 32 oz. plastic trigger spray bottle is recommended for mass application in mark-release-recaptures studies, as outlined in Froerer et al. (2010). Marked flies were then placed in sets of 25 male or 25 female flies per 1.0 liter screened lid container. Four containers of marked flies of each sex and four containers of unmarked flies of each sex, for use as a control, were held in an insectary maintained at 24° C and 70% relative humidity for zero hours, 24 hours, 48 hours, 72 hours, one week, two weeks, three weeks, and five weeks. For flies receiving the zero-hour treatment, individuals were removed shortly after application of the ink. Agar, a 99.6% sucrose sugar cube (C&H Sugar Company, www.chsugar.com), and a protein cake consisting of three parts sucrose, one part protein yeast hydrolysate, and 0.5 parts torula yeast (Lake States Division, Rhinelander Paper Company, Rhinelander, WI) were placed on the top of each screened container. A total of 112 containers were set up for this experiment. At the end of the specified holding periods, the numbers of dead flies were counted and living flies were frozen in order to obtain accurate counts.

To preserve flies, individuals were stored at -80° C until they were processed for marker detectability. Following the final holding time in week five, samples from all holding periods were assessed to determine dye retention. Samples from the different holding times were randomly selected for the order of assessment. Dye retention was determined subjectively by a single observer with qualitative ratings. The dye was detected visually using a black light and a magnifying glass. The number ‘1’ was given if the mark was easy to detect, ‘3’ if the mark was somewhat hard to detect, ‘5’ if the mark was hard to detect.

Because the duration of holding times were chosen to represent well-spaced intervals within the five weeks that the experiment was run, a Mixed Effects Statistical Model was deemed appropriate (Crawley 2002). Sex and treatment were modeled as fixed effects, while time in hours since marking was modeled as a random effect. Differences in proportion of dead flies at the end of each holding time were tested by using *nlme* in R statistical software package (Pinheiro 2008).

**Figure 1.**
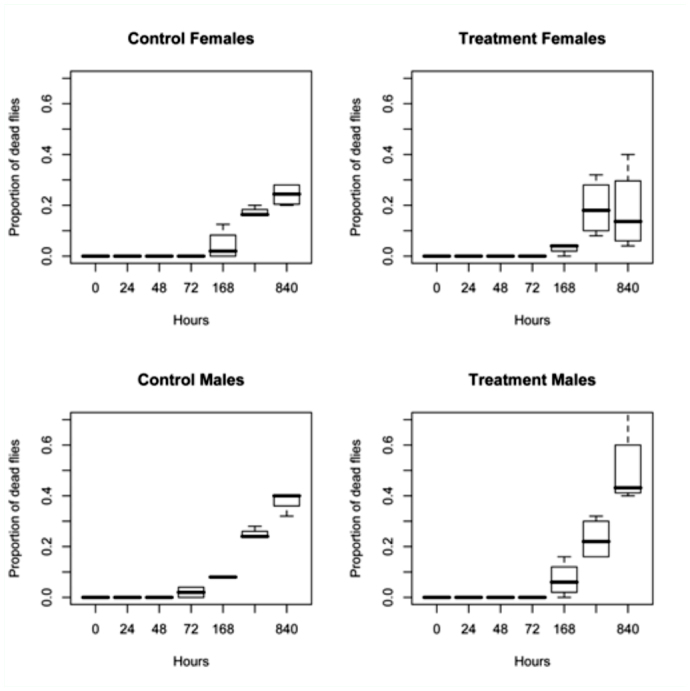
The box plots of the proportion of adult *Bactrocera dorsalis* flies killed at the end of each holding time and control. The females and males are matched for each holding time in order to visually compare the differences in mortality between the controls and treatments. High quality figures are available online.

Finally, in order to examine marker persistence on flies in protein baited traps, an additional group of flies were separated by gender into 3 groups of 25 flies, for a total of 75 males and 75 females. In this non-replicated trial, flies were individually marked and placed in 3, 5-gm torula yeast pellets per 300 ml water and left for 2 or 7 days.

## Results

Overall, the marker had minimal impact on fly survival. The box plots representing percentage survival of treatment and control flies for each holding period is presented in Figure 1. Results from the Mixed Effects Statistical Model indicate no differences between treatment and control mortality (*p* = 0.658) or in the treatment by sex interaction (*p* = 0.465). However, mortality variation was explained by sex, and was statistically significant (*p* < 0.05). Therefore, a second model, AIC model 1, that not only examined random intercepts, but also assumed slope as a random effect and treatment as a fixed effect was a significant improvement over the previous model (AIC model 1 = -258.27 vs. -224.07, *p* < 0.01). Examination of Figure 1 indicates a higher variance in the proportion of flies that died. Among treatment flies, 100% were alive; only two males died among the control group through 72 hours. Weeks 1– 5 show an increase in mortality. However, because increase in mortality is found in both treatment and control flies, natural mortality with increasing age was assumed as a common cause of death.

**Figure 2.**
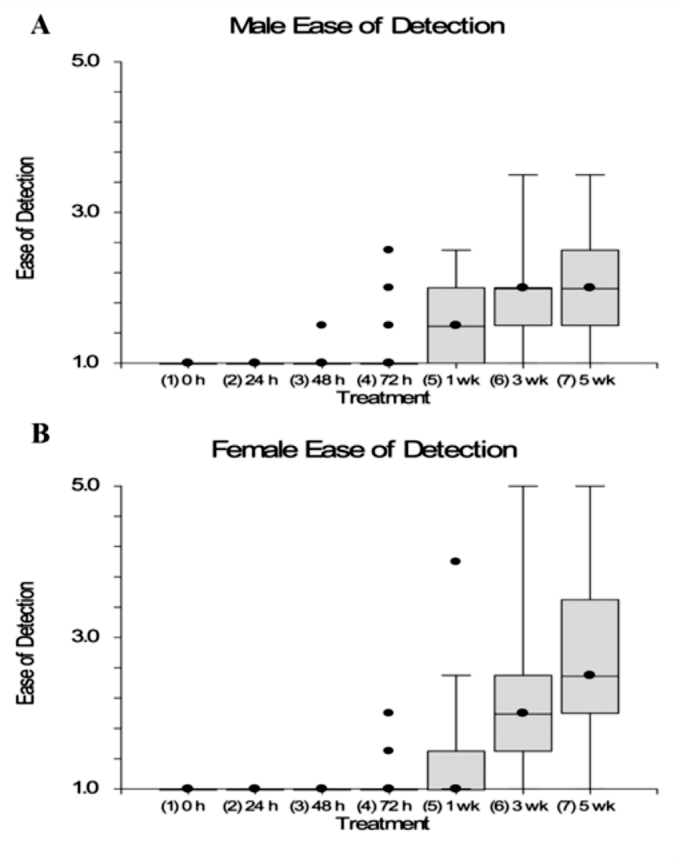
The box plots of the assessment of dye retention on adult *Bactrocera dorsalis* flies for each holding time. Each fly was given a rating for the ease of detection. The number ‘1’ was given if the mark was easy to detect, ‘3’ if the mark was somewhat hard to detect, ‘5’ if the mark was hard to detect. (A) Female; (B) male. High quality figures are available online.

Of 1400 marked flies, only three did not show a visible mark. The ease of detection of the marker for each holding time is presented in Figure 2. Even though the mean detection ratings rose slightly with the last three holding times, averages were only just above that of “easy to detect” score.

The dye on all of the marked flies placed in the torula yeast was detected after two and seven days without exception.

## Discussion

The results show that the readmission ink is an effective marker for the oriental fruit fly. In this experiment, the ink proved to be quick drying, non-toxic to the flies, and showed excellent retention over a five-week period. Marked flies were easily identified under a black light with the aid of a magnifying glass.

Results suggest this marking technique has great potential for future mark-release-recapture dispersal studies for a variety of arthropods. In addition to non-toxicity and long retention rates, the ink is also inexpensive; ∼ $26 USD per 16 fluid ounces, with approximately .09 liters used per 1000 flies. This estimation applies to topical application only, while marking in mass by spraying requires even less dye. The favorable results of this study encouraged the use of the marker in a movement study of the oriental fruit fly during the summer months of 2007 (Froerer et al. 2010). Over 200,000 flies were marked with the readmission ink using the spray technique and released within the Puna District on the island of Hawaii. The flies were released into the natural climatic conditions of a tropical environment where normal behavioral activity such as foraging and mating were possible. Over 1200 flies with easily detected markings were recaptured eight days after release, an additional 200 were recaptured day 11, and 84 were recaptured on day 15 (Froerer et al. 2010). The large number of recaptured flies and the ease of marker-detection demonstrates excellent rigor and retention of the dye in field conditions.

Further tests may establish the potential of this ink for use with other insects. Methyl eugenol traps and liquid protein bait traps were used in the movement study mentioned above. It did not appear that either of the traps affected retention of the readmission ink marker; the flies placed in the torula yeast retained their dye over the course of seven days. Results may differ with varying trapping techniques not yet tested, such as adhesive sticky ball or tape traps. While results from the movement study did not indicate any effects of sunlight, humidity, or precipitation on the marker, further tests concerning these variables will be useful. Further testing is recommended, such as testing mark transfer feasibility during mating and close quarters, and testing of application methods, such as ensuring a thickness that is easy to detect but does not cause increased mortality or impede flight.
